# A novel whole‐brain imaging pipeline for amyloid‐beta plaque integrated with downstream iterative immunolabeling in pre‐clinical Alzheimer’s Disease animal models

**DOI:** 10.1002/alz.090850

**Published:** 2025-01-09

**Authors:** Michael Sasner, Dylan Garceau, Kevin J Elk, Peter Kamelin, Elijah Yew, Ritesh Chidambaram, Tim Ragan

**Affiliations:** ^1^ The Jackson Laboratory, Bar Harbor, ME USA; ^2^ TissueVision Inc., Newton, MA USA; ^3^ TissueVision, Somerville, MA USA

## Abstract

**Background:**

Alzheimer’s Disease (AD) has a strong spatial‐temporal component to its progression, where different brain regions are affected by amyloid‐beta (Aβ) plaque deposition at varying time points and in distinct cell types. Standard imaging and analysis platforms can neglect these details, as they lack the ability to pair high‐yield whole‐brain imaging with region‐specific or high‐resolution analysis. Here we describe a novel high‐throughput whole‐brain imaging pipeline to quantitatively track plaque progression as a function of brain region across time, while also producing indexed tissue sections for secondary staining and analysis that can be registered back to the original brain image.

**Method:**

Aβ plaques in novel knock‐in mouse models of familial AD were labeled with Methoxy‐X04, an Aβ plaque‐specific compound. In some cases, brain vasculature was also labelled with DyLight. The brains were processed on the TissueCyte Serial Two‐Photon Plus (STP+) imaging platform to produce fully aligned multi‐channel volumetric datasets, yielding high resolution 3D models of each brain. Registration to the Allen Mouse Brain Common Coordinate Framework (CCFv3) and region‐specific plaque analysis were conducted to determine plaque size, density, and total number per brain region. By correlating Methoxy and DyLight signals, cerebral amyloid angiopathy (CAA) was also quantified. Indexed brain sections were then used for iterative immunolabeling (IBEX; see Radtke et al, Nat Protocols 2022) to detect vascular and cellular responses to amyloid on representative sections and remapped to the original 3D whole‐brain dataset.

**Result:**

The analysis of whole‐brain plaque distribution in familial AD mouse models revealed distinct spatial‐temporal changes across the brains. STP+ imaging, combined with CCFv3 mapping and secondary analysis of microglial and astrocytic markers allowed for targeted evaluation of Aβ plaques, vascular pathology, and a comparison of regional molecular changes between models.

**Conclusion:**

We demonstrate here that the high sensitivity and precision of the STP+ platform coupled with secondary analysis of cellular responses can facilitate mechanistic understanding of amyloid deposition and cellular responses associated with specific therapeutic approaches. This approach could be very useful to study amyloid‐related imaging abnormalities (ARIA) in preclinical models.

